# Innovative Precision Gene‐Editing Tools in Personalized Cancer Medicine

**DOI:** 10.1002/advs.201902552

**Published:** 2020-04-23

**Authors:** Xiaofeng Dai, Pilar Blancafort, Peiyu Wang, Agustin Sgro, Erik W. Thompson, Kostya (Ken) Ostrikov

**Affiliations:** ^1^ Wuxi School of Medicine Jiangnan University Wuxi 214122 China; ^2^ The Harry Perkins Institute of Medical Research Nedlands Western Australia 6009 Australia; ^3^ School of Human Sciences The University of Western Australia Nedlands Western Australia 6009 Australia; ^4^ The Greehey Children's Cancer Research Institute The University of Texas Health Science Center at San Antonio San Antonio TX 78229 USA; ^5^ Institute of Health and Biomedical Innovation Queensland University of Technology Brisbane Queensland 4059 Australia; ^6^ School of Biomedical Sciences Queensland University of Technology Brisbane Queensland 4059 Australia; ^7^ Translational Research Institute Woolloongabba Queensland 4102 Australia; ^8^ School of Chemistry and Physics Queensland University of Technology Brisbane Queensland 4000 Australia

**Keywords:** CRISPR, genome‐editing, precision medicine, transcription activator‐like effector nucleases (TALENs), zinc finger nucleases (ZFNs)

## Abstract

The development of clustered regularly interspaced short palindromic repeats (CRISPR) has spurred a successive wave of genome‐engineering following zinc finger nucleases and transcription activator‐like effector nucleases, and made gene‐editing a promising strategy in the prevention and treatment of genetic diseases. However, gene‐editing is not widely adopted in clinics due to some technical issues that challenge its safety and efficacy, and the lack of appropriate clinical regulations allowing them to advance toward improved human health without impinging on human ethics. By systematically examining the oncological applications of gene‐editing tools and critical factors challenging their medical translation, genome‐editing has substantial contributions to cancer driver gene discovery, tumor cell epigenome normalization, targeted delivery, cancer animal model establishment, and cancer immunotherapy and prevention in clinics. Gene‐editing tools, epitomized by CRISPR, are predicted to represent a promising strategy toward the precise control of cancer initiation and development. However, some technical problems and ethical concerns are serious issues that need to be appropriately addressed before CRISPR can be incorporated into the next generation of molecular precision medicine. In this light, new technical developments to limit off‐target effects are discussed herein, and the use of gene‐editing approaches for treating otherwise incurable cancers is brought into focus.

## Graphical abstract

Genome‐editing has made substantial contributions to cancer driver gene discovery, tumor cell epigenome normalization, targeted delivery, cancer animal model establishment, and cancer immunotherapy and prevention in clinics. Herein, the current status and promise of gene‐editing tools in clinics are brought into focus, and advances and future perspectives in resolving technical and ethical issues limiting their clinical translation are discussed.

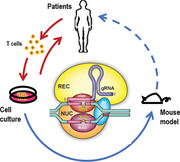

## 1 Introduction

**Figure 1:**
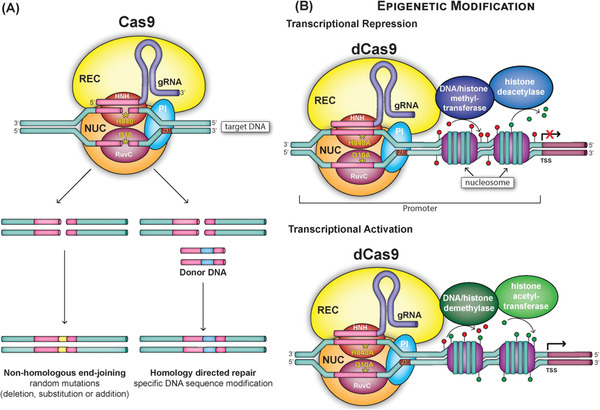
Schematic representation of Cas9 from *Streptococcus pyogenes* (SpyCas9) used for CRISPR‐Cas9 gene‐editing and regulation. A) Cas9 consists of an RNA‐recognition lobe (REC, yellow), which binds the guide RNA (gRNA) molecules and the nuclease lobe (NUC, orange), which in turn contains distinct domains for binding and cleavage of the target DNA. Once a gRNA has bound to the REC lobe, Cas9 is then able to bind the target DNA. The target DNA must contain a sequence complementary to the gRNA on the target strand (shown as a pink tube) as well as a protospacer adjacent motif (PAM) on the nontarget strand. Cas9 interacts with the PAM through the PAM‐interacting (PI) domain located within the NUC lobe of Cas9. This induces melting of the DNA strand, allowing the DNA to be cleaved by the HNH and RuvC nuclease domains, which are also located in the NUC lobe of Cas9. The catalytic residues, H840 of the HNH nuclease domain and D10 of the RuvC nuclease domain, are essential for DNA cleavage. B) Mutation of the catalytic residues of HNH (H840A) and RuvC (D10A) abolishes the nuclease activity of Cas9. This defective Cas9 (dCas9) can then be used to target‐specific DNA sequences without modifying the DNA and may be used to epigenetically regulate the transcription of specific genes. dCas9 can be conjugated with DNA methyltransferases (DNMT) and histone deacetylases (HDAC) to add methyl groups (red lollipops) and remove acetyl groups (green lollipops) from DNA for transcriptional repression; or conjugated to demethylases and histone acetylases (HAT) for transcriptional activation. REC, RNA recognition lobe; NUC, nuclease lobe; gRNA, guide RNA; PI, protein interaction domain; PAM, protospacer adjacent motif; DNMT, DNA methyltransferase; HDAC, histone deacetylase; HAT, histone acetyltransferase; TSS, transcription start site.

CRISPR (clustered regularly interspaced short palindromic repeats) capability has spurred a tsunami of precision gene‐editing during the past few years, following zinc finger nucleases (ZFNs) and transcription activator‐like effector nucleases (TALENs). It has revolutionized the landscape of genome engineering with improved precision and efficiency as well as reduced cost and complexity through introducing DNA double‐strand breaks (DSBs) at the genomic locus of interest, and repair of DSBs by the error‐prone nonhomologous end joining (NHEJ) or homology‐directed repair (HDR) pathways.^[^ ^1^, ^2^ ^]^ CRISPR‐associated systems essentially represent an “adaptive immunity” in prokaryotes, protecting the cells from invading (e.g., phages and extra‐chromosomal plasmid) DNA. Specifically, in the CRISPR/Cas9 system, Cas9 is an endonuclease that forms a complex with a single guide RNA (sgRNA) that is artificially fused from a CRISPR RNA (crRNA) and a *trans*‐activating crRNA (tracrRNA). The sgRNA is designed to recognize a 20‐nucleotide complementary genomic sequence containing a downstream protospacer‐adjacent motif (PAM). The nucleic acid RNA guides Cas9 protein to the targeted genomic locus and cuts DNA to induce a DSB that is approximately three nucleotides upstream of the PAM sequence. This is followed by NHEJ or HDR repair, leading to site‐specific precision gene‐editing. Further modification of Cas9 to abolish catalytic activity (defective Cas9, dCas9) has enabled the development of the technology for the locus‐specific manipulation of the epigenetic state, for epigenome engineering (**Figure** **1**).

**Figure 2:**
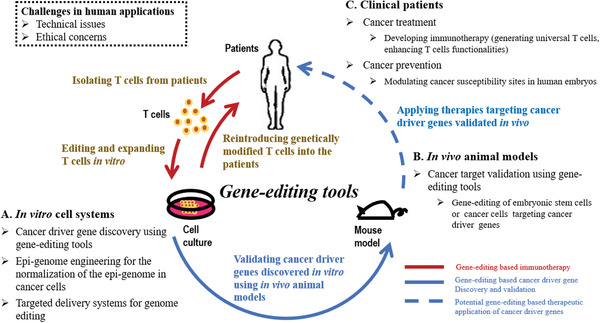
Conceptual scheme illustrating how gene‐editing tools contribute to cancer precision medicine in vitro, in vivo, and in clinics. A) In in vitro cell systems, gene‐editing tools can be used for cancer driver gene discovery. B) In in vivo animal models, gene‐editing tools can be used to establish animal models for cancer target validation. C) For clinical patients, gene‐editing tools can be used to generate universal T cells or enhance T‐cell functionalities during immunotherapy development for cancer treatment, and modulate cancer susceptibility sites in human embryos for cancer prevention. Applying gene‐editing tools in clinics is challenged by technical issues such as off‐target effect and ethical concerns. Many efforts are devoted to resolve these issues that lead the future trend.

The results examined here suggest that establishment of the gene‐editing tools indeed facilitates translation of fundamental knowledge on genome functionalities into the clinic, and, moreover, enabled several important discoveries in personalized cancer medicine. With this in mind, in the following sections we summarize how gene‐editing tools contribute to the three primary stages of cancer precision medicine: cancer driver gene discovery in vitro, tumor animal model establishment in vivo, and cancer management in clinics, with a focus on new technological developments based on CRISPR systems (**Figure** **2**) using a systematic approach (Supporting Information).

## 2 Preclinical Studies Based on Gene‐Editing Tools

### 2.1 Discovery of Cancer Driver Genes Using Gene‐Editing Tools

Large‐scale genomic screening is a powerful technology capable of detecting genes in which mutations drive cancer initiation and progression. Reagents such as short interfering RNAs (siRNAs), short hairpin RNAs (shRNAs) and exogenous plasmids have been widely used to identify and characterize targeted genes through altering their expression; however it is not always possible to achieve site‐specific modulations at the precision of base pairs using these techniques. CRISPR/Cas9 technology provides a rapid approach to modify endogenous loci to overcome these limitations (**Table** **1**). For example, Wang and co‐workers used a pooled lentiviral library (73 000 sgRNAs) to perform a positive selection screen in KBM7 human chronic myelocytic leukemia (CML) cells and HL60 acute myelocytic leukemia (AML) cells, and identified two genes involved in DNA mismatch repair, *TOP2A* and *CDK6*, the loss of which confers resistance to 6‐thioguanine and DNA topoisomerase II poison etoposide, respectively.^[^ ^3^ ^]^ Shalem et al. performed a positive selection screen from a genome‐scale CRISPR/Cas9 knockout library comprising 18 080 genes using a pooled lentiviral delivery of 64 751 unique guide sequences in melanoma cells, and identified a panel of genes including the previously validated *NF1A* and *MED12*, and novel hits *NF2*, *CUL3*, *TADA2B*, and *TADA1*, in which mutations conferred resistance to protein kinase BRAF inhibitor vemurafinib.^[^ ^4^ ^]^

**Table 1:** Example applications of epigenome and genome‐editing for preclinical cancer control

Study	Organism	Disease	Stage	Editing tool	Section	Application
Zhang et al., 2011, *Nat. Biotechnol*.	Human	NA	In vitro	TALE	Epigenome engineering	Gene activation using VP64
Provasi et al., 2012, *Nat. Med*.	Human	NA	In vitro	ZFN	Cancer treatment	Generation of universal T cells for cancer immunotherapy
Torikai et al., 2012, *Blood*	Human	NA	In vitro	ZFN	Cancer treatment	Generation of universal T cells for cancer immunotherapy
Stolzenburg et al., 2012, *Nucleic Acids Res*.	Human	Breast cancer	In vitro	ZF	Epigenome engineering	Gene repression using SKD repressor domain
Torikai et al., 2013, *Blood*	Human	NA	In vitro	CRISPR/Cas9	Cancer treatment	Generation of universal T cells for cancer immunotherapy
Wang et al., 2013, *Cell*	Mouse	NA	In vivo	CRISPR/Cas9	Animal model establishment	Establishment of GEMM
Yang et al., 2013, *Cell*	Mouse	NA	In vivo	CRISPR/Cas9	Animal model establishment	Establishment of GEMM
Hwang et al., 2013, *Nat. Biotechnol*.	Zebrafish	NA	In vivo	CRISPR/Cas9	Animal model establishment	Gene expression modulation in Cas9‐expressing animal models
Friedland et al., 2013, *Nat. Methods*	Nematode	NA	In vivo	CRISPR/Cas9	Animal model establishment	Gene expression modulation in Cas9‐expressing animal models
Gilbert et al., 2013, *Cell*	Human	NA	In vitro	CRISPR/dCas9	Epigenome engineering	Gene activation using VP16
Hai et al., 2014, *Cell Res*.	Pig	NA	In vivo	CRISPR/Cas9	Animal model establishment	Gene expression modulation in Cas9‐expressing animal models
Xue et al., 2014, *Nature*	Mouse	Liver cancer	In vivo	CRISPR/Cas9	Animal model establishment	Establishment of nGEMM
Sanchez‐Rivera et al., 2014, *Nature*	Mouse	Lung cancer	In vivo	CRISPR/Cas9	Animal model establishment	Establishment of nGEMM
Maddalo et al., 2014, *Nature*	Mouse	NA	In vivo	CRISPR/Cas9	Animal model establishment	Establishment of nGEMM
	Mouse	Lung cancer	In vivo	CRISPR/Cas9	Animal model establishment	Gene expression modulation in Cas9‐expressing animal models
Niu et al., 2014, *Cell*	Monkey	NA	In vivo	CRISPR/Cas9	Animal model establishment	Gene expression modulation in Cas9‐expressing animal models
Findlay et al., 2014, *Nature*	Human	NA	In vitro	CRISPR/Cas9	Cancer driver gene discovery	Focused LOF genetic screen against particular pathways
Wang et al., 2014, *Science*	Human	CML, AML	In vitro	CRISPR/Cas9	Cancer driver gene discovery	Large‐scale LOF genetic screen
Shalem et al., 2014, *Science*	Human	Melanoma	In vitro	CRISPR/Cas9	Cancer driver gene discovery	Large‐scale LOF genetic screen
Gilbert et al., 2014, *Cell*	Human	CML	In vitro	CRISPR/dCas9	Cancer driver gene discovery	Large‐scale GOF genetic screen
Wang et al., 2015, *Science*	Human	CML	In vitro	CRISPR/Cas9	Cancer driver gene discovery	Focused LOF genetic screen against particular pathways
Birsoy et al., 2015, *Cell*	Human	T‐ALL	In vitro	CRISPR/Cas9	Cancer driver gene discovery	Focused LOF genetic screen against particular pathways
Stolzenburg et al., 2015, *Oncogene*	Mouse	Breast cancer	In vivo	ZF	Epigenome engineering	Gene repression via DNA methylation using DNMT3A
Thakore et al., 2015, *Nat. Methods*	Human	Leukemia	In vitro	CRISPR/dCas9	Epigenome engineering	Gene repression using KRAB
Konermann et al., 2015, *Nature*	Human	Melanoma	In vitro	CRISPR/dCas9	Cancer driver gene discovery	Large‐scale GOF genetic screen
Poirot et al., 2016, *Cancer Res*.	Human	NA	In vitro	TALEN	Cancer treatment	Generation of universal T cells for cancer immunotherapy
Castro et al., 2016, *Drug Future*	Human	NA	In vitro	CRISPR/Cas9	Cancer treatment	Generation of universal T cells for cancer immunotherapy
Chew et al., 2016, *Nat. Methods*	Mouse	NA	In vivo	CRISPR/Cas9	Targeted delivery system	CRISPR system package and delivery using AAV‐based multifunctional platform
Garcia‐Bloj et al., 2016, *Oncotarget*	Human	Lung cancer	In vitro	CRISPR/dCas9, ZF, TALE	Epigenome engineering	Gene activation (tumor suppressor gene reactivation) using VP64, p300, VPR, SAM
Amabile et al., 2016, *Cell*	Human	NA	In vitro	CRISPR/dCas9, TALE	Epigenome engineering	Gene repression using KRAB and via DNA methylation using DNMT3A
Xu et al., 2016, *Cell Discovery*	Human	NA	In vitro	CRISPR/dCas9	Epigenome engineering	Gene activation via DNA demethylation using TET1
Cano‐Rodriguez et al., 2016, *Nat. Commun*.	Human	NA	In vitro	CRISPR/dCas9	Epigenome engineering	Gene activation (gene reactivation) via epigenetic modulation using H3K4me3
Liu et al., 2016, *Cell*	Mouse	NA	In vivo	CRISPR/dCas9	Epigenome engineering	Gene repression via DNA methylation using DNMT3A and gene activation via DNA demethylation using TET1
Qasim et al., 2017, *Sci. Transl. Med*.	Human	NA	In vitro	TALEN	Cancer treatment	Generation of universal T cells for cancer immunotherapy
Wang et al., 2017, *Cell*	Human	AML	In vitro	CRISPR/Cas9	Cancer driver gene discovery	Focused LOF genetic screen against particular pathways
Kong et al., 2017, *Nature*	Human	Melanoma	In vitro	CRISPR/Cas9	Cancer driver gene discovery	Focused LOF genetic screen against particular pathways
Drost et al., 2017, *Science*	Human	NA	In vitro	CRISPR/Cas9	Cancer driver gene discovery	Study the origin of cancer mutational signatures
Ren et al., 2017, *Oncotarget*	Human	NA	In vitro	CRISPR/Cas9	Cancer treatment	Generation of universal T cells for cancer immunotherapy
Ren et al., 2017, *Clin. Cancer Res*.	Mouse	NA	In vivo	CRISPR/Cas9	Cancer treatment	Generation of universal T cells for cancer immunotherapy
Ren et al., 2017, *Oncotarget*	Mouse	B‐ALL	In vivo	CRISPR/Cas9	Cancer treatment	Enhancement of T‐cell functionalities in cancer immunotherapy
Liang et al., 2017, *Biomaterials*	Human	Osteosarcoma	In vitro	CRISPR/Cas9	Targeted delivery system	CRISPR system package and delivery using PEG–PEI–cholesterol lipopolymer
Chen et al., 2017, *Adv. Funct. Mater*.	Mouse	Glioma	In vivo	CRISPR/Cas9	Targeted delivery system	CRISPR system package and delivery using liposome‐templated hydrogel nanoparticles
Wang et al., 2017, *Adv. Sci*.	Mouse	Melanoma	In vivo	CRISPR/Cas9	Targeted delivery system	CRISPR system package and delivery using gold nanocluster/lipid core–shell nanocarrier
Liu et al., 2017, *J. Microbiol. Biotechnol*.	Human	NA	In vitro	CRISPR/dCas9	Epigenome engineering	Gene activation using SAM
Klann et al., 2017, *Nat. Biotechnol*.	Human	NA	In vitro	CRISPR/dCas9	Epigenome engineering	Gene activation using p300 and gene suppression using KRAB
Zhou et al., 2018, *Angew. Chem., Int. Ed*.	Human, mouse	NA	In vitro, in vivo	CRISPR/Cas9	Targeted delivery system	CRISPR system loading and delivery using black phosphorus nanosheets
Pflueger et al., 2018, *Genome Res*.	Human	NA	In vitro	CRISPR/dCas9‐SunTag	Epigenome engineering	Gene repression via DNA methylation using DNMT3A with low off‐target activity
Morita et al., 2018, *Methods Mol. Biol*.	Human	NA	In vitro	CRISPR/dCas9‐SunTag	Epigenome engineering	Gene activation via DNA demethylation using TET1
Zhang et al., 2018, *Protein Cell*	Human	NA	In vitro	CRISPR/dCpf1‐SunTag	Epigenome engineering	Gene activation using p300
Guo et al., 2019, *Proc. Natl. Acad. Sci. USA*	Human, mouse	TNBC	In vitro, in vivo	CRISPR/Cas9	Targeted delivery system	CRISPR system package and delivery using nanolipogel system
Li et al., 2019, *J. Controlled Release*	Mouse	Pancreatic cancer	In vivo	CRISPR/Cas9	Targeted delivery system	CRISPR system package and delivery using R8‐dGR peptide modified cationic liposome
Rui et al., 2019, *Sci. Adv*.	Mouse	Glioma	In vivo	CRISPR/Cas9	Targeted delivery system	CRISPR system package and delivery using carboxylated branched poly (*β*‐amino ester) nanoparticles
Liu et al., 2019, *Adv. Sci*.	Mouse	Breast cancer	In vivo	CRISPR/dCas9	Targeted delivery system	Gene activation (miR‐524) via VP64 using multistage delivery nanoparticle (MDNP)
Kretzman et al., 2019, *Chem. Sci*.	Mouse	Breast cancer	In vivo	CRISPR/dCas9 activators	Targeted delivery system	Gene activation (tumor suppressor gene reactivation) via VP64, VPR, SAM and delivery using RGD dendritic polymeric nanoparticles

AML: acute myelocytic leukemia; AAV: adeno‐associated virus; B‐ALL: B‐cell acute lymphoblastic leukemia; CML: chronic myelocytic leukemia; DNMT3A: DNA methyltransferase 3A; GEMM: genetically engineered mouse model; GOF: gain‐of‐function; KRAB: The Krüppel associated box domain; LOF: loss‐of‐function; NA: not available, i.e., not related to any diseases or referring to diseases in general; p300: histone acetyltransferase; PEG: polyethylene glycol; PEI: polyethylenimine; SAM: Synergistic Activation mediato; SKD: KRAB Domain; TALEN: transcription activator‐like effector nuclease; TCR: endogenous T‐cell receptor; T‐ALL: T‐acute lymphoblastic leukemia; TET1: Ten‐eleven translocation methylcytosine dioxygenase 1; TNBC: triple negative breast cancer; VPR; VP64‐p65‐Rta; nGEMM: nongermline genetically engineered mouse model; ZFN: Zinc‐Finger nuclease

Focused genetic screens against particular pathways have also proven fruitful in identifying cancer cell dependencies (Table 1). Wang et al. generated a gene essentiality dataset across 14 human AML cell lines through a genome‐wide CRISPR‐based screen, and proposed a general strategy for defining mammalian gene networks and synthetic lethal interactions.^[^ ^5^ ^]^ Through an unbiased CRISPR/Cas9 knockout screen on melanoma cells that were either resistant or addicted to BRAF inhibition, Kong et al. uncovered a signaling pathway comprised of ERK2 kinase, JUNB, and FRA1 transcription factors that underpins tumor cell addiction to BRAF inhibition.^[^ ^6^ ^]^ Birsoy et al. performed a genetic screen using ≈30 000 sgRNAs to target ≈3000 candidate metabolic pathway players, and observed that loss of *GOT1* (the cytosolic aspartate aminotransferase) could sensitize human Jurkat leukemic T cells to the mitochondrial complex I inhibitor phenformin.^[^ ^7^ ^]^ The Shendure group studied the functionalities of cell‐essential genes to achieve a high‐resolution understanding of regulatory and protein‐coding nucleic acid sequences of interest using HEK293T cells, where CRISPR/Cas9 RNA‐guided cleavage and multiplex HDR were coupled using a complex library of donor templates. In this study, they replaced a 6 bp genomic region in exon 18 of *BRCA1* with all possible hexamers, or the full exon with all possible single nucleotide variants (SNVs), and measured strong effects on transcript abundance attributable to nonsense‐mediated decay and exonic splicing elements. Similarly, they performed saturation genome‐editing of a well‐conserved coding region of *DBR1*, and measured the relative phenotypic consequences.^[^ ^8^ ^]^

CRISPR‐modified human stem‐cell‐derived organoids have also been used to explore the origin of cancer mutational signatures (Table 1). Using the CRISPR/Cas9 technique to delete key DNA repair genes from human colon organoids, followed by delayed subcloning and whole‐genome sequencing, Drost et al. revealed that mutation accumulation in organoids deficient in *MLH1* is driven by replication errors, and that the mutational footprint (signature 30) can arise from germline *NTHL1* mutations.^[^ ^9^ ^]^

Besides the loss‐of‐function (LOF) screens using the Cas9 nuclease, dCas9 has been utilized to perform parallel genome‐wide activation screens (Table 1). Gilbert et al. established genome‐scale CRISPR platforms including CRISPRa (activators to endogenous genes constructed using dCas9), with which they identified essential genes for tumor growth and elucidated the mechanisms of cells’ sensitivity to a cholera–diphtheria toxin in CML cells K562.^[^ ^10^ ^]^ Konermann et al. reported structure‐guided genetic engineering of a CRISPR/Cas9 complex to mediate efficient transcriptional activation at endogenous loci. Using this new activation system, they activated endogenous genes and noncoding RNAs, elucidated the rules for effective sgRNA design, established the platform for genome‐wide dCas9‐based transcription activation screen, and applied it in a drug resistance study of melanoma,^[^ ^11^ ^]^ which collectively demonstrated the broad applicability of CRISPR‐based gain‐of‐function (GOF) screen in functional genomics research.

We can thus conclude that the above results collectively demonstrate the broad applicability of CRISPR‐based gain‐of‐function screen in functional genomics research. We further emphasize that the potential power of such efficient genome‐wide gene‐editing systems continue to drive innovative development of new variations and specializations, which no doubt will continue in near future.

### 2.2 Targeted Delivery Systems for Genome and Epigenome‐Editing

The CRISPR/Cas9 system has been combined with various delivery systems taking advantages of both nanotechnologies (nonviral delivery systems) and viruses for enhanced cytosolic delivery (Table 1). For example, a biodegradable 2D delivery platform for precision genome‐editing was established through loading black phosphorus (also known as the fourth phosphorus allotrope, phosphorene) nanosheets with Cas9 ribonucleoprotein (RNP).^[^ ^12^ ^]^ In nanotechnology‐enabled CRISPR/Cas9 delivery systems, it is crucial to balance the biocompatibility of the nanoparticle (NP)‐based delivery vehicles, the ability of the vehicles to release the genetic payload at specified locations, and effective internalization without causing harm to the cells. Requirement of specific sites for the delivery necessitated engineering of the black phosphorus nanosheets with three nuclear localization signals (NLSs), which ensured the success of the cytosolic delivery and release mode of the CRISPR/Cas9 payload in the above example. An osteosarcoma cell‐specific, aptamer‐functionalized PEG–PEI–cholesterol lipopolymer that encapsulates plasmids encoding vascular endothelial growth factor‐A (VEGFA) sgRNA and Cas9 was established to achieve targeted delivery of the CRISPR/Cas9 system for therapeutic genome‐editing of VEGFA.^[^ ^13^ ^]^ Similarly, polymeric dendritic NPs targeted with RGD peptides encapsulating CRISPR/Cas9 activators were effectively targeted to breast tumors in an animal model of breast cancer, resulting in effective tumor suppression.^[^ ^14^ ^]^ A tumor‐targeted nanolipogel system was also demonstrated as a safe, precise and effective delivery approach for in vivo CRISPR genome‐editing by successfully knocking out lipocalin 2 (Lcn2) in triple negative breast cancers (TNBC),^[^ ^15^ ^]^ and a tumor‐targeted lipid‐based CRISPR/Cas9 delivery system was established by co‐encapsulating plasmids encoding Cas9, sgRNA targeting Hypoxia‐inducible factor 1‐α (HIF‐1α), and paclitaxel (PTX) that conveys antimetastatic effects, into R8‐dGR peptide‐modified cationic liposomes. The resultant R8‐dGR‐Lip/PTX/pHIF‐1*α* successfully downregulated HIF‐1*α* and its downstream molecules to elevate the antitumor efficacy of PTX.^[^ ^16^ ^]^ An adeno‐associated virus (AAV)‐based multifunctional platform, named AAV‐split‐Cas9, which is efficient in delivery yet does not induce extensive cellular damage in vivo, was also customized for genome‐editing.^[^ ^17^ ^]^

With regard to epigenetic editing, SadCas9 fused to KRAB has been used for long‐term gene silencing of *PCSK9* in mice. *PCSK9* is a hepatic gene responsible for cholesterol homeostasis. Gene repression and low density lipoprotein (LDL) cholesterol levels were maintained for up to 24 weeks after a single systemic administration of AAV8 dual‐vector, CRISPR, and the gRNA, in postmitotic cells.^[^ ^18^ ^]^ SadCas9‐2xVP64 and sgRNAs were also delivered via AAV9 to treat muscular dystrophy, by upregulating in a mutation‐independent fashion the compensatory gene *LAMA‐1* in a mouse model of congenital muscular dystrophy type 1A,^[^ ^19^ ^]^ and the AAV9 system was also exploited to deliver Sp dCas9 and an optimized truncated 14 bp dgRNA MS2 RNA aptamer to recruit MS2‐p65‐HSF1 transcriptional activator to treat mouse models of muscular dystrophy, acute kidney disease, and diabetes.^[^ ^20^ ^]^ Additionally, AAV9 effectively transduced Cas9 and gRNA in muscle stem cells and repaired the mutated dystrophin gene in dystrophic mdx mice. Genome‐editing in stem cells holds promise for a more sustained gene repair therapy for the monogenic disorder Duchenne muscular dystrophy (DMD).^[^ ^21^ ^]^ Improved muscle histology has been achieved 6–8 weeks after systemic delivery of AAV9‐Cas9 in a dog model of DMD;^[^ ^22^ ^]^ however, long‐term (1 year) dystrophin protein restoration via AAV8/9‐Cas9 genome‐editing in adult mdx mice led to humoral and cellular immune responses as well as induced unwanted off‐target effects, which requires further studies.^[^ ^23^ ^]^

Various artificial viruses utilizing the “core–shell” structure of viruses have also been constructed for targeted delivery of CRISPR/Cas9 system in vivo. For instance, an artificial virus was established consisting of a core of fluorinated polymer (PF33) binding with the CRISPR/Cas9 system and a versatile shell comprising a multifunctional polymer obtained by modifying natural hyaluronan (HA) polymer with PEG side chains and the R8‐RGD tandem peptide; it showed efficiency for targeted genome‐editing in mice in terms of loading with the CRISPR/Cas9 system, accelerating endosomal escape, and promoting nuclear penetration without additional nuclear‐localization signals.^[^ ^24^ ^]^ A multistage delivery nanoparticle (MDNP) was designed as a core–shell structure, where the core is a cationic polyplex made from CRISPR/dCas9 plasmid DNA and phenylboronic‐acid‐modified low‐molecular‐weight polyethyleneimine, and the shell is made of a responsive polymer (2,3‐dimethylmaleic anhydride‐modified poly(ethylene glycol)‐*b*‐polylysine) that allows MDNP to overcome multiple physiological barriers and achieve targeted delivery of dCas9 and miRNA‐524 to tumors in vivo.^[^ ^25^ ^]^

It is worth noting that AAV delivery for CRISPR systems has the potential of unattended effects due to the integration of the viruses, while nonviral delivery approaches may minimize these effects. Another issue is immunogenicity elicited by the viral proteins and the nucleic acids. In cancer, too, there is a limitation as far as the availability of targeted viruses that are engineered to have appropriate tropism. The nonviral approaches partially circumvent these issues with the flexibility of scaffolds and the targeted ligands that can be engineered, and the PEGylation and other methods to make the particles immune inert. Since cytosolic plasmid DNA can induce host immune responses,^[^ ^26^ ^]^ an efficient and safer alternative is the development of cationic lipid‐mediated CRISPR‐Cas9 RNPs for gene‐editing.^[^ ^27^ ^]^ Several RNP scaffolds have been developed for targeted delivery of CRISPR gene‐editing tools. A preclinical example is the amelioration of hearing loss in the Beethoven mouse model carrying a type of autosomal‐dominant form of progressive deafness.^[^ ^28^ ^]^ Cas9 RNPs have been also encapsulated in biodegradable glutathione‐cleavable nanocapsules and locally administered to achieve robust gene‐editing in skeletal muscle and retinal tissue in vivo with reduced cytotoxicity.^[^ ^29^ ^]^ Further, Cas9 RNPs enabled gene‐editing in an orthotopic glioma mouse model after a single intracranial administration of self‐assembling carboxylated branched poly(*β*‐amino ester) nanoparticles, with efficient endosomal escape.^[^ ^30^ ^]^ Finally, targeted delivery of Cas9 RNPs using liposome‐templated hydrogel nanoparticles (LHNPs) effectively inhibited tumor growth and increased survival in tumor‐bearing mouse. Additionally, LHNPs can be exploited to treat brain tumors if engineered with an autocatalytic brain‐tumor‐targeting (ABTT) mechanism.^[^ ^31^ ^]^ This approach relies on a positive feedback loop by stimulating blood–brain barrier (BBB) modulators, thus increasing NP delivery via transcytosis or through the BBB gaps.^[^ ^32^ ^]^ Gold nanoclusters based on a shell of polyethylene glycol–lipid (LGCP) represent another nanotechnology platform for Cas9‐RNP delivery, which suppressed melanoma progression in mice.^[^ ^33^ ^]^ Gold NPs assembled with cationic endosomal disruptive polymers have been locally injected into a mouse model of DMD. The delivery of Cas9 RNP effectively corrected the DNA mutation without substantial off‐target effects.^[^ ^34^ ^]^ Lastly, dCas9 protein fused to VPR has been delivered by the Genome‐editing with Designed Extracellular Vesicles (GEDEX) system. Extracellular vesicles (EVs), produced and shed by HEK293 cells, were able to co‐deliver dCas9‐VPR protein and sgRNAs and upregulate hepatocyte growth factor (HGF), the growth factor essential for liver regeneration, in a mouse model of liver damage.^[^ ^35^ ^]^

Analysis of the above CRISPR‐based targeted delivery systems validates their potential as a foundation for the establishment of effective genome therapeutics, which, in our opinion, are particularly effective for the various nonviral options. Further development and testing of these is highly warranted, and we foresee opportunities in inducing host immunogenicity, and would like to emphasize the possibility of long‐term side effects in patients.

### 2.3 Establishment of Animal Models for Cancer Target Validation Using Gene‐Editing Tools

CRISPR/Cas9 can largely reduce the cost and time needed to establish genetically engineered mouse models (GEMMs) and nongermline GEMMs (nGEMMs) of cancer (Table 1). For example, Wang et al. developed a strategy to disrupt multiple genes in mouse embryonic stem (ES) cells in a single step.^[^ ^36^ ^]^ They generated mice harboring bi‐allelic mutations in *Tet1* and *Tet2* with an efficiency of 80% by co‐transfecting Cas9 mRNA and sgRNAs targeting both genes into zygotes, and achieved the simultaneous disruption of five genes (*Tet1*, *Tet2*, *Tet3*, *Sry*, and *Uty*) without significantly sacrificing efficiency.^[^ ^36^ ^]^ Using CRISPR/Cas9, Yang et al. successfully generated mouse models carrying a tag or a fluorescent reporter construct in the *Nanog*, *Sox2*, and *Oct4* genes, and established *Mecp2* conditional mutant mice in one step using mouse ES cells.^[^ ^37^ ^]^

CRISPR/Cas9 systems can be applied to adult mice, which can considerably reduce the time required for GEMM and nGEMM establishment and provide a much easier approach to generate multiple gain‐of‐function and loss‐of‐function animal models (Table 1). Xue et al. directly injected DNA plasmid encoding Cas9 and sgRNAs that target *p53* and *Pten* (two tumor suppressor genes) into mouse liver and induced tumor growth.^[^ ^38^ ^]^ Meanwhile, Ventura and co‐workers demonstrated the feasibility of adapting the CRISPR/Cas9 system to model large oncogenic chromosomal rearrangements in wild‐type mice via an *Eml4‐Alk* inversion through the delivery of an adenovirus encoding Cas9 and two sgRNAs.^[^ ^39^ ^]^

CRISPR/Cas9 systems allow easy and flexible genome modulations in animal model studies where targeting the germline used to be a great challenge (Table 1). Platt et al. established a mouse model with stable Cas9 expression, which can be utilized to modulate the genetic information in different organs of adult mice models using the CRISPR/Cas9 system.^[^ ^40^ ^]^ It is therefore not surprising that quite similar approaches have been applied in other species including zebrafish,^[^ ^41^ ^]^ *Caenorhabditis elegans*,^[^ ^42^ ^]^ pigs,^[^ ^43^ ^]^ and cynomologus monkeys.^[^ ^3^ ^]^

### 2.4 EpiGenome Engineering in Cancer Cells

Unlike changes of sequence promoted by CRISPR/Cas9 systems, epigenetic editing is reversible, and thus offers an opportunity to “reverse” multigene programs in carcinoma cells by normalizing gene expression “at will.” Epigenetic inhibitors, such as DNA methyltransferases and histone deacetylase inhibitors, approved for cancer treatment, lack locus selectivity, whereas epigenetic editing enables local and precise targeted reconfiguration of the chromatin. This opens the door to functional epigenomics to determine the causative role of particular modifications in chromatin and distinguish “driving” versus “passenger” effects during oncogenesis and cancer progression.

Epigenetic editing methods exploit a sequence‐specific engineered DNA‐binding domain linked to one or more epigenetic activities (Table 1).^[^ ^44^ ^]^ Pioneering tools with zinc finger (ZF) domains demonstrated that six ZF arrays linked to either transcriptional activators or with DNA demethylase domains (e.g., TET1, TET2 catalytic domains) are able to overcome the epigenetic silencing state of genes marked by DNA methylation, such as mammary tumor suppressors^[^ ^45^, ^46^ ^]^ and candidate tumor suppressor genes.^[^ ^47^ ^]^ Initially TALEs and dCas9 backbones were linked to single transactivators such as VP16,^[^ ^48^, ^49^ ^]^ and to epigenetic activities associated with gene activation such as histone acetyltransferase p300.^[^ ^50^ ^]^ To enhance the potency of gene activation, current approaches recruit multiple activator domains to the same genomic location with the dCas9‐SunTag system (where SunTag is a protein scaffold that can recruit multiple copies of an antibody‐fusion protein),^[^ ^51^ ^]^ consisting of ten copies of a GCN4 peptide interspaced by a 5 amino acid linker. Epigenetic recruitment is mediated by anti‐GCN4 scFv fused to a given epigenetic domain, and this technology has been broadly applied for targeted gene activation^[^ ^52^ ^]^ and editing of DNA methylation in human cells.^[^ ^53^ ^]^

To mitigate the shortcomings of the methods discussed above, an interesting alternative strategy exploits arrays of multiple epigenetic activities, for example, by engineering CRISPR‐associated RNA scaffolds, such as aptameric sequences recognizing the bacteriophage coat protein MS2 (Table 1).^[^ ^11^ ^]^ This system has been recently applied to the upregulation of the human gamma interferon promoter, which enhanced innate immunity and decreased tumorigenesis,^[^ ^34^ ^]^ and also to strong reactivation of tumor suppressors silenced by DNA methylation.^[^ ^54^ ^]^ A similar approach has been adapted for CRISPR‐dCas9‐mediated DNA demethylation, by fusing MS2 with TET1.^[^ ^55^ ^]^ In addition to DNA demethylation, the engineering of novel epigenetic catalytic activities, such as those inducing H3K4me and H3K79me, have the potential to increase the longevity of the activation state in silenced tumor suppressor genes marked by DNA methylation.^[^ ^56^ ^]^

Similar to gene re‐activation, epigenetic repressor domains have been engineered with ZF, TALEs, and dCas9 systems (Table 1).^[^ ^57^ ^]^ While the KRAB domain represents a potent transcriptional suppressor, it has transient effects.^[^ ^58^ ^]^ In contrast, linkage of engineered binding proteins with the catalytic domain of DNA methyltransferases, such as mammalian DNA methyltransferase 3A (DNMT3A)/B, promoted DNA methylation editing in the targeted genomic sequences. We showed that 6ZFs‐DNMT3A fusions designed against the promoter and enhancer of the *SOX2* transcription factor in breast cancer were associated with a window of epigenetic and transcriptional memory in a mouse model of breast cancer.^[^ ^59^, ^60^ ^]^ Liu et al. described precise editing of DNA methylation with dCas9‐DNMT3A fusions in mammalian cells which blocked CCCTC‐binding factor (CTCF) binding.^[^ ^61^ ^]^ To minimize off‐target effects, some strategies have exploited the SunTag system to enhance the efficiency and specificity of targeted methylation.^[^ ^62^ ^]^

Current, and in our opinion most promising, approaches for epigenetic silencing are based on the linkage of multiple domains that reinforce the repressive effect of the epigenetic editing potentially with long‐lasting effects (Table 1). For example, co‐delivery of DNMT3A, KRAB, and DNMT3L (a co‐factor of DNMT3A) fused with ZFs, TALEs, or dCas9 resulted in sustainable repression of targeted genes in mammalian cells with maintenance of DNA methylation in a “hit‐and‐run” approach.^[^ ^63^ ^]^ It is important to acknowledge, however, that the context dependence of the epigenome at the targeted regulatory regions may require very specific combination of epigenetic effector domains for effective epigenome normalization. As more mechanistic knowledge is integrated in the field of functional epigenetics, we believe that it may be possible, in the future, to dissect pre‐emptive rules for assembly of combination of domains for targeted epigenetic engineering of eventually any regulatory region of the genome.

### 2.5 Clinical Applications of Gene‐Editing Tools

Ever since the first CRISPR/Cas 9 clinical study posted in 2015 by the Children's Research Institute, USA, that targeted the *NF1* gene in tumors of the central nervous system, more and more CRISPR/Cas 9 clinical studies have been launched. Of those currently recorded in the ClinicalTrials.Gov website, 1 study was initiated in 2015, 4 in 2016, 5 in 2017, 9 in 2018, 5 in 2019 and 2 in 2020, out of which 15 are based in China, 10 in USA, and 1 in France (**Table** **2**).

**Table 2:** Example applications of gene‐editing tools for clinical cancer control

NCT Number	Status	Editing tool	Target	Disease	Country	Group	Phase	Actual study start date
NCT02793856	Active, not yet recruiting	CRISPR	PD‐1	Metastatic non‐small cell lung cancer	China	Sichuan University	Phase 1	26‐Aug‐16
NCT02863913	Withdrawn	CRISPR	PD‐1	Muscle‐invasive bladder cancer	China	Peking University	Phase 1	1‐Sep‐16
NCT02867332	Withdrawn	CRISPR	PD‐1	Metastatic renal cell carcinoma	China	Wujiang Liu, Peking University	Phase 1	1‐Nov‐16
NCT02867345	Withdrawn	CRISPR	PD‐1	Castration resistant prostate cancer	China	Wujiang Liu, Peking University	Phase I	1‐Nov‐16
NCT03044743	Recruiting	CRISPR	PD‐1	Advanced stage EBV associated malignancies	China	Nanjing University Medical School	Phase I/II	7‐Apr‐17
NCT03057912	Unknown	TALEN; CRISPR/Cas9	HPV16 E6/E7T1 or HPV18 E6/E7T2	Human papillomavirus‐related malignant neoplasm	China	First Affiliated Hospital, Sun Yat‐Sen University	Phase 1	20‐Feb‐17
NCT03081715	Completed	CRISPR	PD‐1	Advanced esophageal squamous cell carcinoma	China	Hangzhou Cancer Hospital	Phase 1	11‐Mar‐17
NCT03166878	Recruiting	CRISPR	TCR/B2M	B‐cell leukemia/lymphoma	China	Chinese PLA General Hospital	Phase I/II	1‐Jun‐17
NCT03332030	Suspended	CRISPR	NF1	Neurofibromatosis type 1, tumors of the central nervous system	USA	Children's Research Institute	Unknown	27‐Nov‐15
NCT03398967	Recruiting	CRISPR	TCR	B‐cell leukemia/lymphoma	China	Chinese PLA General Hospital	Phase I/II	5‐Sep‐18
NCT03399448	Active, not yet recruiting	CRISPR	TCR/PD‐1	Multiple myeloma, melanoma, synovial sarcoma, myxoid/round cell liposarcoma	USA	University of Pennsylvania	Phase 1	5‐Sep‐18
NCT03538613	Withdrawn	CRISPR	CISH	Metastatic gastrointestinal epithelial cancer	USA	National Cancer Institute	Phase I/II	17‐Mar‐18
NCT03545815	Recruiting	CRISPR	TCR/PD‐1	Mesothelin positive multiple solid tumors	China	Chinese PLA General Hospital	Phase I	1‐Jun‐18
NCT03606486	Recruiting	Crispr‐Duplex sequencing	TP53 mutations	Ovarian carcinomas	USA	University of Washington	Unknown	16‐Nov‐18
NCT03690011	Not yet recruiting	CRISPR	CD7.CAR/28zeta	T‐cell acute lymphoblastic leukemia/lymphoma	USA	Baylor College of Medicine	Phase 1	1‐Mar‐20
NCT03747965	Recruiting	CRISPR	PD‐1	Adult solid tumor	China	Chinese PLA General Hospital	Phase 1	1‐Nov‐18
NCT04035434	Recruiting	CRISPR	Unknown	Relapsed or refractory B‐cell malignancies	USA	CRISPR Therapeutics AG	Phase I/II	22‐Jul‐19
NCT04037566	Recruiting	CRISPR	HPK1	Relapsed or refractory haematopoietic malignancies	China	Xijing Hospital	Phase 1	1‐Aug‐19

Among these studies, 18 trials are associated with cancers, which include virus‐induced malignancies such as advanced stage Epstein‐Barr virus (EBV)‐associated malignancies (NCT03044743, 2017, China) and human papilloma virus (HPV)‐related malignant neoplasm (NCT03057912, 2017, China), leukemias such as B‐cell leukemia/lymphoma (NCT03398967, 2018, China; NCT03690011, 2017, China; NCT04035434, 2019, US), T‐cell acute lymphoblastic leukemia/lymphoma (NCT03690011, 2020, US), relapsed or refractory hematopoietic malignancies (NCT04037566, 2019, China), and solid tumors such as metastatic gastrointestinal epithelial cancer (NCT03538613, 2018, US), neurofibromatosis type 1 (NF1) tumors of the central nervous system (NCT03332030, 2015, US), and T‐cell receptor (TCR)/programmed cell death 1 (PD‐1) in multiple myeloma, melanoma, synovial sarcoma, myxoid/round cell liposarcoma (NCT03399448, 2018, US). Currently, all these clinical studies are in phase I or phase I/II to test the safety, tolerability and efficacy of CRISPR gene‐editing products except for the study sponsored by Hangzhou Cancer Hospital in China (NCT03081715, 2017, China) that has completed the clinical phase I stage and reported no safety concerns on the use of *PD‐1* knockout engineered T cells in treating advanced oesophageal cancers.^[^ ^64^ ^]^ Approximately 90% of these phase I/II clinical studies were estimated to be completed in 2020 or the following 2 years, and successful regimens would be carried over to larger scales of clinical investigations before the wide applications of gene‐editing tools in clinics.

### 2.6 Applications of Gene‐Editing Tools in Cancer Treatment Generation of Universal T Cells for Cancer Immunotherapy

CRISPR/Cas9 has been proposed to bypass the host rejection of cell therapies by eliminating human leucocyte antigen (HLA) expressed on the surface of allogeneic T cells,^[^ ^65^ ^]^ and been used to generate universal chimeric antigen receptor‐T (CAR‐T) cells to allow increased numbers of patients to access immunotherapies. Qasim et al. generated universal CAR19 (chimeric antigen receptor against the B‐cell antigen CD19) T cells through lentiviral transduction of nonhuman leukocyte antigen‐matched donor cells and simultaneous TALEN‐mediated gene‐editing of the T‐cell receptor and *CD52* gene loci in 2017.^[^ ^66^ ^]^ They administered a single‐dose infusion of universal CAR19 T cells to two infants carrying relapsed refractory CD19^+^ B‐cell acute lymphoblastic leukemia (B‐ALL) following lymphodepleting chemotherapy and anti‐CD52 serotherapy, and observed successful molecular remission ahead of allogeneic stem cell transplantation.^[^ ^66^ ^]^ Ren et al. generated allogeneic CAR‐T cells deficient of endogenous TCR, HLA class I molecule and PD1 through lentiviral CAR delivery and CRISPR RNA electroporation, and showed that disrupting checkpoint molecules can add to the in vivo antitumor efficacy of CRISPR‐modified CAR‐T cells.^[^ ^67^ ^]^ CRISPR Therapeutics launched a clinical trial (CT) on its phase I candidate CTX110 (NCT04035434) which is an off‐the‐shelf CRISPR‐engineered CAR‐T‐cell therapy targeting CD19 in relapsed or refractory B‐cell malignancies.

### 2.7 Enhancement of T Cells’ Functionalities in Cancer Immunotherapy

The efficiency of TCR and CAR‐T cells in immunotherapy can be thwarted by immunosuppression from both tumor and tumor microenvironment (Table 2). Some inhibitory receptors expressed on T cells, such as cytotoxic T‐lymphocyte antigen 4 (CTLA‐4), PD‐1, domain‐containing protein‐3 (TIM3), and lymphocyte‐activated gene‐3 (LAG‐3) can be activated by certain ligands expressed on the tumor cell surface to terminate T‐cell activation and dampen immune responses. Their encoding genes can be ablated through genome‐editing to enhance the functionalities of CAR‐T cells.^[^ ^68^, ^69^, ^70^ ^]^ Referred to as “checkpoint blockade,” such therapies have demonstrated great success in both hematologic malignancies and solid tumors,^[^ ^71^ ^]^ where gene‐editing technologies play significant roles. For example, the CRISPR/Cas9 system has been used to achieve high‐efficiency editing of multiple genes including those encoding HLA, PD‐1 and CTLA‐4, by combining several sgRNAs in one manipulation to generate exhaustion‐resistant T cells.^[^ ^72^ ^]^ Ren et al. disrupted *PD‐1* in CD19 CAR‐T cells using TCR and HLA‐I double knockout mice and demonstrated enhanced antileukemia activity in a xenograft model of B‐ALL, suggesting that genetic disruption of checkpoint molecules can enhance CAR‐T‐cell activity.^[^ ^73^ ^]^ Although capable of potently re‐activating antitumor T cells, checkpoint inhibitors can also lead to uncontrolled proliferation of T cells and severe autoimmunity in patients.^[^ ^74^ ^]^

In late 2016, a man in China with aggressive lung cancer who had failed all other therapeutics became the first person receiving CRISPR‐based therapy. In this clinical case, CRISPR/Cas9 was used to knock out the gene encoding PD‐1 before immune cell expansion and infusion back into the patient. Expansion of this clinical trial to more patients and in more types of cancers began in April 2017 (NCT03044743).^[^ ^75^ ^]^ Similarly, an immunotherapy trial utilizing the CRISPR technique to target both PD‐1 and the endogenous T‐cell receptor within engineered CAR‐T cells in melanoma patients was conducted at the University of Pennsylvania (NCT03399448) in 2018, and is ongoing.^[^ ^75^, ^76^ ^]^ A similar trial was carried out by the Lu group at Sichuan University in China (NCT02793856), while Peking University plans to initiate three clinical trials of *PD1*‐knockout in autologous T cells using CRISPR/Cas9 system against bladder‐, prostate‐, and renal‐cell cancers.^[^ ^77^ ^]^

The outcomes of these trials further substantiate the importance of genome engineering in the clinic, particularly in the field of cancer immune therapy, marking a major milestone in the field of genome‐editing. However, the apparent limitation of off‐target effects leading to partial inconsistency in validation and cross‐referencing of the results from different studies apparently represents interesting future opportunities.

### 2.8 Editing of Cancer Driver Genes in Cancer Therapy

The unique nature of genomic rearrangements due to genomic instability in cancers provides an opportunity for cancer‐specific genes, and carries a potentially lower ethical burden, especially in consideration of otherwise incurable cancers. The first affiliated hospital of Sun Yat‐Sen University initiated a clinical trial in 2017 to assess the safety and efficacy of TALEN and CRISPR/Cas9 in treating human cervical intraepithelial neoplasia I without invasion via targeting HPV16 and HPV18 E6/E7 DNA that drives HPV persistency (NCT03057912). Xijing hospital, also from China, posted a clinical trial to edit endogenous *HPK1* in patients carrying relapsed or refractory CD19^+^ leukemia or lymphoma using the CRISPR technique in 2019 which is currently recruiting (NCT04037566). Though most clinical trials on gene‐editing focus on immune cells with a large proportion targeting on *PD‐1*, efforts need to be and will be expanded to more genes with cancer driving roles as our knowledge on their functionalities and potential side effects continue to grow.

### 2.9 Applications of Gene‐Editing Tools in Cancer Prevention

Besides all aforementioned applications of gene‐editing tools in somatic cells, CRISPR/Cas9 can potentially be used to correct germline pathogenic gene mutations or modulate tumor susceptibility sites in human embryos to reduce the risk of developing genetic diseases (Table 2). Existing examples include correcting the heterozygous *MYBPC3* mutation in human preimplantation embryos to prevent hypertrophic cardiomyopathy (HCM),^[^ ^74^ ^]^ replacing mutations in *HTT* genes with the correct sequence to reverse Huntington's disease immune dysfunction,^[^ ^78^ ^]^ editing mutant *APP* in human fibroblasts to reduce the risk of developing early‐onset Alzheimer's diseases,^[^ ^79^ ^]^ and coding a mutation in *CCR5* in human embryos to prevent cholera, smallpox, and acquired immunodeficiency syndrome (AIDS).^[^ ^80^ ^]^ Though the CRISPR technique has not been applied to prevent cancers, a hypothetical example would be to edit cancer predisposition genes *BRCA1/2* in human embryos to prevent breast and ovarian cancers once ethically permitted, and a clinical trial screening mutations in p53 using CRISPR‐Duplex sequencing with the aim of identifying women with high risk of developing ovarian cancers for early diagnosis was initiated in 2018 and is ongoing (NCT03606486). Even though gene‐editing tools are potentially feasible for preventing cancer occurrence by correcting heritable mutations in human embryos, we emphasize that, if improperly used, serious ethical concerns to both academia and society may arise (see below), thus necessitating a well‐defined and effective regulatory framework.

## 3 Challenges Faced by Gene‐Editing Tools and Developing Solutions

### 3.1 Technical Issues

Restricted genomic target sites as imposed by sgRNA design need to be resolved before this technology can become the first‐line approach in personalized cancer medicine. Ongoing efforts have been devoted to discover new principles for sgRNA design with enhanced Cas9 activities and improved target efficiency. Hsu et al. found that sgRNAs with +67 or +85 nucleotides tracrRNA tails mediated the most efficient Cas9 cleavage, and two mismatches, either concatenated or interspaced, could significantly reduce the activity of Cas9, especially if they occurred in the region adjacent to the PAM region.^[^ ^81^ ^]^ Others have been focusing on exploring the feasibility of using evolutionarily divergent Cas9 proteins, such as *Streptococcus thermophilus* Cas9 (St1Cas9),^[^ ^82^ ^]^ *Neisseria meningitides* Cas9 (NmCas9),^[^ ^83^ ^]^ *Staphylococcus aureus* Cas9 (SaCas9),^[^ ^84^ ^]^ and *Streptococcus* Cas9 (SpCas9),^[^ ^85^ ^]^ for genome engineering, given the high presence of antibodies against these Cas9 orthologs in adult human population.^[^ ^85^, ^86^, ^87^ ^]^ These orthologs have different PAM recognition sequences and enzyme activities,^[^ ^82^ ^]^ enriching our toolbox for precision gene‐editing.

CRISPR/Cas9 as a genome‐editing tool for gene therapy induces p53 activation due to DSB repair,^[^ ^88^, ^89^ ^]^ which may be advantageous for treating cancers harboring p53 mutation, but otherwise reduces the Indel generation efficiency in cells. CRISPR/Cas‐mediated single‐base editing represents an alternative approach to genome‐editing in the absence of a DNA‐repair template without generating DSBs.^[^ ^90^ ^]^ This new strategy may be particularly efficient in postmitotic cells with reduced HDR activity^[^ ^91^ ^]^ or to avoid activation of p53‐mediated DNA‐repair machinery as a consequence of induced double‐stranded DNA cleavage that severely reduces the genome‐editing efficiency.^[^ ^88^, ^89^ ^]^ The single base editors (BE) consist of either a nickase (D10A) SpCas9 (nCas9) or a catalytically dead (D10A/H840A) SpCas9 (dCas9) that is N‐terminally fused to deaminases. Examples are the cytidine deaminase base editor (CBE) comprising the rat or human APOBEC1/3A,^[^ ^90^ ^]^ and the tRNA adenine deaminase base editor (ABE).^[^ ^92^ ^]^ Collectively these enzymes enable the programmable conversion of C:G to T:A and A:T to G:C, respectively, with low rates of nonspecific insertions, deletions, or other mutations (indels). However, since single base conversion editing can be repaired by the endogenous base excision repair (BER) pathways, second‐generation editors Sp and Sa nCas9‐APOBEC1 fusions have been engineered with two C‐terminal copies of the uracil glycosylase inhibitor (UGI) that inhibits this repair.^[^ ^93^ ^]^ Further, codon usage optimization and modifications in the localization of the NLS in both CBEs and ABEs have substantially increased the efficiency of single base pair editing.^[^ ^94^ ^]^ Importantly, substantial genome‐wide off‐target effects, mostly SNVs, appear to be induced by engineered CBEs but to a lesser extent by ABEs.^[^ ^95^, ^96^ ^]^ To overcome the restriction of nCas9 in recognizing G/C‐rich PAM sequences, APOBEC1 has been linked to dCas12a *Lachnospiraceae bacterium* LbdCpf1 to target canonical T‐rich PAM sites.^[^ ^97^ ^]^ Alternatively, an enhanced version of the *Acidaminococcus sp* AsdCas12a enables recognition of noncanonical T‐rich PAM sequences.^[^ ^98^ ^]^ Human APOBEC3A‐nCas9 fusions are particularly critical for targeting hypermethylated genomic regions where the rat APOBEC1 is relatively inefficient.^[^ ^99^ ^]^ The human APOBEC3A also enables the recognition of 5–17 bases in the target DNA, in contrast with the rat enzyme recognizing only 5 bps.^[^ ^100^ ^]^ Further, an enhanced version of the human APOBEC3A has been engineered to reduce unwanted base editing when multiple cytidines are present within the cognate motif of the *β*‐thalassemia gene promoter.^[^ ^101^ ^]^

Off‐target effects may cause undesired mutations at random sites which, if occurring in evolutionarily constrained regions of the human genome or overlapping with epigenetic signals, may compromise precision and in vivo safety, and individual heterogeneity such as single nucleotide variants could considerably affect the efficacy and consequence of genome‐editing. Thus, maximization of genomic specificity is critical to minimize the risk of producing or selectively expanding cells harboring undesired pathologically relevant mutations^[^ ^102^ ^]^ for clinical genome‐editing. This can be approached by using high‐fidelity variants of dCas9,^[^ ^103^ ^]^ by adapting new dCas proteins such as Cas12a (dCas12a),^[^ ^104^, ^105^ ^]^ or by modifying the sgRNAs to enhance specificity.^[^ ^106^ ^]^ Experimental techniques such as high‐throughput genome‐wide, translocation sequencing (HTGTS),^[^ ^107^ ^]^ breaks labeling and enrichment on streptavidin and sequencing (BLESS) or breaks labeling in situ and sequencing (BLISS),^[^ ^108^, ^109^ ^]^ genome‐wide, unbiased identification of DSBs enabled by sequencing (GUIDE‐seq),^[^ ^110^ ^]^ integrase‐defective lentiviral vectors (IDLV) capture,^[^ ^111^ ^]^ circularization for in vitro reporting of cleavage effects by sequencing (CIRCLE‐seq),^[^ ^112^ ^]^ Digenome‐seq,^[^ ^113^ ^]^ and selective enrichment tagged genomic DNA ends sequencing (SITE‐seq)^[^ ^114^ ^]^ have been established to examine the genome‐wide modification landscape after genome‐editing. Several studies have demonstrated the importance of optimizing sgRNA design in reducing the off‐target effect.^[^ ^115^ ^]^ Computational tools such as Cas‐OFFinder^[^ ^116^ ^]^ and in vivo off‐target quantification strategy (i.e., VIVO) were established to assist in the appropriate design of sgRNAs toward precise genome‐editing.^[^ ^117^ ^]^ In epigenome‐editing, we clearly see a research need to determine how the technology can be tailored to the specific epigenetic contexts, in which case the armamentarium of multiple domains may have to be developed to fully normalize the epigenome. Additionally, as multiple epidomains are engineered in fusion with protein backbones, the delivery of such molecular complexes becomes a greater challenge, particularly for the editing of highly heterogeneous tumors. The engineering of orthologous, more compact variants of dCas9, such as *S. aureus* dCas9, could facilitate the programmable delivery of epigenetic editing tools in vivo.^[^ ^118^ ^]^

Researchers and practitioners should bear in mind that the establishment and use of appropriate delivery systems are vital to avoid potential other side effects in genome‐editing. Transient transfection of the heterologous plasmids has achieved great success and been widely applied for in vitro studies; however, this may induce host cell immune responses in vivo, considerably impeding the clinical translation of such genome‐editing tools. Viral vectors such as adenoviral vectors (AdVs),^[^ ^119^ ^]^ recombinant adeno‐associated viral vectors,^[^ ^120^ ^]^ and IDLVs^[^ ^121^ ^]^ have been employed to achieve stable transfections of the CRISPR system into mammalian cells.

It is worthwhile to distinguish somatic and germline gene‐editing approaches. While enhancement of CAR‐T‐cell therapies through genome‐editing is promising and already employed in several clinical trials both in USA and China (see above and Table 2), other somatic gene‐editing cancer therapies are limited by the need to target essentially all tumor cells, and all cells that do not receive the desired modification will retain their growth advantage and tumorigenic capability, imposing one fundamental challenge of somatic cancer therapies. Germline gene‐editing therapies can ensure genome modification in all cells; however, any mistake such as off‐target effect once occurred will be inherited to all cells and be detrimental or even embryonically lethal.

### 3.2 Ethical Concerns

On 26 November 2018, one day before the Second International Human Genome Editorial Summit, Dr. Jiankui He from China's Southern University of Science and Technology announced the birth of two “CRISPR babies” who should have acquired natural resistance to cholera, smallpox, and AIDS through receiving CRISPR/Cas9 genome‐editing on the *CCR5* gene. This was the first clinical report of human manipulation for disease prevention worldwide, but raised serious ethical queries in both academia and society and ultimately resulted in his recent incarceration.

Indeed, there are several ethical concerns for gene‐editing tools. First, gene‐editing will lead to permanent genome modulation, and will cause serious health problems to the patients as well as future generations if any mistakes occur, either in the design or by accident, during the editing process. Second, despite all the technological developments described above, gene‐editing tools such as CRISPR are not yet fully mature approaches for precise genetic editing, and should not be applied in humans before more advanced generations become available. Third, CRISPR can enable us, in principle, to modulate any genome at will, which may generate huge social conflicts and inequities if it was overused or abused. Moreover, society needs to gradually adapt to changes brought by gene‐editing tools. Lander et al., in their full moratorium on clinical uses of human reproductive editing, proposed that no clinical application of germline gene‐editing should be considered unless its long‐term biological consequences are sufficiently understood both for individuals and for the human species, and “genetic correction” that could have a beneficial effect should be clearly distinguished from “genetic enhancement” that may violate human equality.^[^ ^122^ ^]^ This moratorium does not apply to somatic gene‐editing such as somatic editing of CAR‐T cells and treating sickle cell diseases which are regulated just like traditional gene therapies in the USA, and does not apply to germline gene‐editing for research uses as long as genetically modified embryos are not transferred to a person's uterus. CRISPR‐mediated editing or regulated expression of cancer‐specific driver gene mutations may also be considered as a special circumstance with respect to gene‐editing, due to their targets; however, this will depend on advances in, and acceptability of, mechanisms to deliver gene‐editing machinery to tumor cells to both primary and metastatic sites, as described above.

## 4 Conclusions

The great success of using gene‐editing tools in cancer control preclinically, as detailed herein, has made a new generation of precision medicine possible. Consecutive preclinical and clinical successes have led to a plethora of CRISPR‐related innovations, several of which have now advanced to clinical testing. Though ethical concerns require careful use of genome‐editing tools and may limit its clinical applications, they may not all apply in cancer management for global health benefits. The potential for clinical utility of gene‐editing is reflected in a number of biotech companies based around these platforms, including CRISPR Therapeutics, Editas Medicine, and Intellia Therapeutics.

CRISPR systems, being the third generation of gene‐editing tools, have offered us extreme precision and convenience in genome modulation. However, advances in resolving technical issues, primarily off‐target effects, need to be achieved before it can be applied safely in human. Several derivatives of CRISPR techniques were established to either solve the aforementioned problems and/or extend the functionalities of gene‐editing tools in disease control. For example, the Casilio system is comprised of the dCas9 protein and an sgRNA appended with one or more Poly(U)‐binding‐splicing factor (PUF)‐binding site(s) (sgRNA‐PBS),^[^ ^123^ ^]^ allowing multiplexing gene‐editing in different directions. More endonucleases with similar or enhanced features are being discovered to enrich the toolbox for gene‐editing, such as Cas12a which is a sgRNA‐guided endonuclease matured from Cas12a‐containing CRISPR arrays without recruiting tracrRNA,^[^ ^52^ ^]^ and Cas13 enzymes (Cas13a, Cas13b, Cas13c) that are class 2 type VI RNA‐targeting CRISPR system effectors used for RNA‐editing.^[^ ^124^ ^]^ A novel technology (LEAPER—leveraging endogenous ADAR for programmable editing of RNA) works similarly to CRISPR/Cas13 but relies on just one component known as arRNA to perform RNA editing, and was recently reported to offer an easier deliverable and safer tool regarding the potentially generated undesirable cellular immune response as compared with the double‐component module (guide RNA plus enzyme).^[^ ^125^ ^]^ Emerging endonucleases as such make it possible to achieve multiple types of tasks in one cell when coupled with the CRISPR system and/or alike. Combinations of CRISPR systems with other genetic modulation tools such as Cre/LoxP^[^ ^126^ ^]^ to enable various inducible genome‐editing systems^[^ ^127^ ^]^ further enhance the precision and flexibility of gene‐editing and enrich the toolbox. On the other hand, CRISPR/Cas9‐based “signal conductors” have been created by including modified riboswitches to sgRNAs that can be used to reprogram the fate of cancer cells.^[^ ^128^ ^]^ Significant efforts to increase the ethical and safety acceptances of these approaches are also needed, and emphasis should be placed on the unique nature of malignancy‐related targets to deliver the ultimate promises of personalized medicine.

By combining gene‐editing systems with other molecular functionality tools, we may largely extend the flexibility and scope of what we can achieve in cancer management. Cold atmospheric plasma (CAP), being generated by high‐voltage electrical discharges in atmospheric pressure air and composed of reactive oxygen and nitrogen species, may aid in CRISPR precision medicine by facilitating CRISPR system delivery and creating synergies with CRISPR‐mediated cancer driver gene targeting, epigenome normalization and cancer immunotherapy. The efficacy of CAP‐mediated plasmid delivery has been reported both in vitro^[^ ^129^ ^]^ and in vivo;^[^ ^130^, ^131^ ^]^ and the use of CAP‐activated air in delivering plasmid DNA in a 3D human skin model provides a noncontact DNA transfer platform in vivo,^[^ ^130^ ^]^ which may resolve the biocompatibility problem of the nanoparticle‐based delivery vehicles. The confirmed selectivity of CAP on various types of cancer cells,^[^ ^132^, ^133^ ^]^ efficacies on epigenome modulation,^[^ ^134^, ^135^, ^136^ ^]^ and capability of inducing immunogenic cell death^[^ ^137^ ^]^ all suggest potential synergies between CAP and CRISPR in precision oncotherapies that warrant extensive studies. Efforts in these directions will accelerate the speed of translating genome‐editing tools to precision therapies, and will continue to grow.

## Conflict of Interest

The authors declare no conflict of interest.

## Supporting information

Supporting Information
