# Supplementary material for: Innovative Precision Gene‐Editing Tools in Personalized Cancer Medicine
Source: Adv Sci (Weinh). 2020 Apr 23;7(12):1902552. doi: 10.1002/advs.201902552 (PMC7312441; doi:10.1002/advs.201902552)
Supplement: Supplementary file 1 — Supporting Information [file ADVS-7-1902552-s001.pdf]

Copyright WILEY-VCH Verlag GmbH & Co. KGaA, 69469 Weinheim, Germany, 2020.

Supporting Information

Title: Innovative precision gene editing tools in personalized cancer medicine

*Xiaofeng Dai *, Blancafort Pilar, Peiyu Wang , Agustin Sgro, Erik W Thompson, Kostya (Ken) Ostrikov*

Systematic literature searching strategy

Below we present a systematic review of pre-clinical and clinical studies employing gene editing approaches.

1. Eligibility criteria

Studies eligible for this review included original research papers and clinical trials reports that were published in peer-reviewed journals and/or on the ClinicalTrials.Gov website, and assessed gene editing tools for personalized cancer medicine. “Participants” (P) include people who are pathologically diagnosed with cancer, 18 years to 70 years (adult, older adult), expected life span over 6 months, major organs functioning normally, and willing and able to provide informed consent. With ECOG performance status of 0-2. “Exposure” (E) was considered as CRISPR/Cas9 gene therapy or ZFN- or TALEN-mediated gene editing T cell receptor therapy. “Outcomes” (O) were primary outcome measures and secondary outcome measures. Primary outcome measures employed in these studies included, variably, Response Rate, Number of patients with dose limiting toxicity, Study of related adverse events, Maximum tolerated dose (MTD) and Copy numbers of CAR in peripheral blood (PB), bone

marrow (BM) and lymph nodes. Secondary outcome measures were Disease Control Rate (DCR), Progression free survival (PFS), Overall Survival (OS), Peripheral blood circulating tumor DNA, Peripheral blood T lymphocyte subsets, and/or Incidence of Treatment-Emergent Adverse Events (the time frame is from 3 month to 2 years).

2. Search strategy

The Preferred Reporting Items for Systematic Reviews and Meta-Analysis (PRISMA) guidelines were followed where applicable. An electronic search of Medline (using the Ovid interface), Web of Science databases, and website https://clinicaltrials.gov last updated on 05 February 2020, was performed. Titles (field) of journal articles, published in English (language), were searched for two elements, with several (keywords) indicative of each element combined using the Boolean operators; (i) cancer (cancer* OR carcinoma* OR tumour* OR tumor* OR neoplasm*), AND (ii) Gene editing tools (CRISPR-edited genes therapy* OR CRISPR/Cas9*) AND (TALEN* OR transcription activator-like effector nucleases*) AND (ZFNs* OR zinc finger nucleases*).

3. Study selection

All study citations identified from the original search were imported to Endnote library. Duplicates from overlapping database search, and common key words were removed. Titles and abstracts of the remaining articles were screened for eligibility, and articles for full-text review were identified, and eligible articles were selected. Bibliographies of selected articles were then screened, which resulted in additional eligible articles.

4. Data abstraction

Data selected for gene editing tool in preclinical studies are included in **Table 1**. Data common to this Table include Study, Organism, Disease, Stage, Editing tool, Section, and Application. Data selected for gene editing in clinical trials are included in **Table 2**. Data common to this Table include NCT Number, Status, Editing tool, Target, Disease, Country, Group, Phase, and Actual study start date.
